# Evaluation of adenosine deaminase activity in serum of cattle and buffaloes in the diagnosis of bovine tuberculosis

**DOI:** 10.14202/vetworld.2020.110-113

**Published:** 2020-01-13

**Authors:** Navdeep Kaur Dhaliwal, Deepti Narang, Mudit Chandra, Gursimran Filia, Sikh Tejinder Singh

**Affiliations:** Department of Veterinary Microbiology, College of Veterinary Sciences, Guru Angad Dev Veterinary and Animal Sciences University, Ludhiana, Punjab, India

**Keywords:** adenosine deaminase, comparative intradermal tuberculin test, interferon-γ, *Mycobacterium bovis*

## Abstract

**Background and Aim:**

Bovine tuberculosis (bTB) is a chronic bacterial disease of cattle caused by *Mycobacterium bovis*. bTB causes severe economic losses resulting from livestock deaths, chronic disease, and trade restrictions. Determination of serum levels of adenosine deaminase (ADA), an enzyme produced by monocytes/macrophages and lymphocytes, has been used in the diagnosis of human TB. This study aimed to evaluate the role of ADA enzyme activity in the diagnosis of bTB.

**Materials and Methods:**

In this study, a total of 100 animals (cattle and buffaloes) were screened for bTB by comparative intradermal tuberculin test (CITT) and interferon-γ (IFN-γ) test and in serum samples obtained from 100 screened animals, ADA seric activity was evaluated using ADA-MTB kit procured from Tulip Diagnostics.

**Results:**

A total of 18 animals were positive TB reactors by CITT, 8 were positive by IFN-γ, and 4 animals were positive by both CITT and IFN-γ. The average ADA value of bTB-positive animals either by CITT, IFN-γ, or both CITT and IFN-γ was 12.55 U/L, 14.8 U/L, and 18.36 U/L, respectively, in CID negative, it was 10.57 U/L and in IFN-γ negative, it was 10.59 U/L.

**Conclusion:**

The average ADA value of bTB-positive animals positive either by CITT, IFN-γ, or both CITT and IFN-γ was more than the average ADA value in animals negative for bTB by either of the tests.

## Introduction

Bovine tuberculosis (bTB) is caused by *Mycobacterium bovis* which belongs to *Mycobacterium tuberculosis* complex (MTC), which is a globally distributed zoonotic disease in cattle [1]. The MTC includes *M. bovis*, *M. tuberculosis*, *Mycobacterium africanum*, *Mycobacterium microti*, *Mycobacterium canettii*, and *Mycobacterium caprae* [2-4]. The primary route of the transmission of *M. bovis* among all species is respiratory route while other routes include oral, congenital, or even enter through open wounds [5]. bTB is a chronic granulomatous caseous-necrotizing inflammatory disease that mainly affects the lungs and their draining lymph nodes, however, it can also affect other organs depending on the route of infection [6,7]. In developing countries, bTB is enzootic, has an impact on human health, and causes huge economic losses to the animal industry [8]. In conjunction with possessing the largest population of cattle in the world (nearly 300 million cows and buffaloes) (Basic Animal Husbandry and Fisheries Statistics, Government of India 2017), India’s lack of a control program poses a potential threat for bTB infection and its transmission worldwide. The diagnosis of bovine TB depends on clinical manifestations, incidental necropsy evidence, tuberculin skin testing and culture isolation methods, etc. In addition, lymphocyte proliferation, gamma-interferon (IFN-γ) assay, and indirect enzyme-linked immunosorbent assay [9] are also available.

Adenosine deaminase (ADA) is an enzyme that catalyzes hydrolytic deamination of either adenosine or deoxyadenosine to produce inosine and deoxyinosine, respectively. First, the presence of enzyme was detected in whole blood and serum by Conway and Cooke. In domestic animals, ADA is present in all organs, although the highest activity has been found in lymphoid tissues [10]. ADA activity is increased in various conditions such as liver disease, TB, typhoid, infective mononucleosis, and certain malignancies, especially those of hematopoietic origin [11]. The principle of ADA assay is based on the detection of either hydrogen peroxide or ammonia after enzymatic deamination of adenosine to inosine by ADA. ADA is essential for the proliferation and differentiation of lymphoid cells, especially T-cells, and helps in the maturation of monocytes to macrophages.

Intradermal tuberculin test is recognized by the World Organization for Animal Health (OIE) as the primary screening test for the detection of bovine TB in cattle [12]. However, because tuberculin testing is not very sensitive, the development of more sensitive and specific diagnostic tools could improve bovine TB control and eradication programs. Determination of seric levels of ADA has been used in the diagnosis of human TB.

This study aimed to investigate the diagnostic value of serum ADA in diagnosis of bovine TB because rapid and accurate diagnosis is an important element of bTB treatment and control.

## Materials and Methods

### Ethical approval

This study was approved by the Animal Ethics Committee of Guru Angad Dev Veterinary and Animal Sciences University (GADVASU), India.

### Study animals

A total of 100 animals from a herd with the previous history of TB were screened by Comparative intradermal tuberculin test (CITT) and interferon-γ (IFN-γ) assay. A total of 22 TB-positive reactors were selected based on reactivity to CITT and IFN-γ assay while 78 animals were negative by either of the screening tests. Blood samples were collected from all of these 100 animals and serum was separated. Serum samples were stored at 4°C until further use. ADA activity was measured within 48 h of blood collection using ADA kit (Tulip Diagnostics).

### ADA assay procedure

All the reagents and samples were brought to room temperature before use. Working phenol reagent and working hypochlorite reagent were prepared. Both phenol reagent and hypochlorite reagent were diluted 1:5 with distilled water before use (1 part of reagent + 4 parts of distilled water). The spectrophotometer filter was set at 600 nm (Hg at 35-37°C).

Clean dry test tubes were labeled as blank (B), standard (S), sample blank (SB), and test (T). A 0.2 ml of buffer reagent was added in the blank and standard test tube and 0.2 ml of adenosine reagent was added in test and standard blank test tubes. Then, 0.02 ml of deionized water was added in the test tube labeled as blank and 0.02 ml of standard was added in the test tube labeled as standard and 0.02 ml sample was added in the test tube labeled as T.

Mixing was done and test tubes were incubated at 37°C for 60 min. One milliliter of phenol reagent was added in all the four test tubes. A 0.02 ml sample was added in the standard blank test tube and then 1 ml of hypochlorite reagent was added in all the four test tubes. Mixing was done and test tubes were incubated at 37°C for 15 min or at room temperature for 30 min. The absorbance of the Blank (Abs. B), Standard (Abs. S), Sample Blank, (Abs. SB) and Test (Abs. T) against distilled water was measured.

Calculations were done using the following formula:


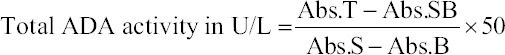


## Results and Discussion

ADA is an enzyme that increases in TB due to the stimulation of T-cell lymphocytes by mycobacterial antigens. ADA has been developed and widely used for the diagnosis of TB due to its simplicity, low cost, and quickly available results [13]. The increase of ADA seric activity in biological fluids has been used with good sensitivity and specificity as an auxiliary diagnostic tool in human TB. Studies have confirmed increase in ADA in TB pericardial effusions, peritoneum, and central nervous system [14-16]. The main reason for the increased ADA levels in pleural effusion is the movement of T-lymphocytes toward this area. Increase in ADA levels is the result of a topical inflammatory reaction caused by monocytes and macrophages [13,16]. When alveolar macrophages are infected by *Mycobacterium*, this enzyme could be found in serum during active pulmonary disease.

In the present study, mean ADA values were 12.55 U/L, 14.8 U/L, and 18.36 U/L in bTB-positive animals either by CITT, IFN-γ, or both CITT and IFN-γ, respectively. The average ADA value in animals negative for bTB by either of the tests was 10.57 U/L (Tables-1 and 2). In positive animals either by CID or IFN-γ, ADA average was found more than 12 U/L. The results of the present study are in agreement with those of Rodrigues *et al*. [17], who reported that ADA seric activity was significantly higher in bTB-infected cattle. They reported a higher than 15 U/L ADA seric value in 10 animals with TB. In maximum animals which were TB positive and negative either by CITT and IFN-γ, ADA values ranged from 10 to 20 U/L. The reason for high serum levels of ADA might be associated with some other diseases such as typhoid fever, infectious mononucleosis, liver disease, sarcoidosis, leukemia, brucellosis, or in other diseases where cellular immunity is stimulated.

**Table-1:** Average values of adenosine deaminase in cattle and buffaloes.

	CID Positive	CID Negative	IFN γ CID Positive	IFN γ Positive
Average	12.55 U/L	10.57U/L	18.36U/L	14.8U/L
Range	4.81-39.05	1.79-26.6	4.81-39.05	4.81-39.05

**Table-2:** ADA levels in cattle and buffaloes tested for bTB by CITT and IFN-γ assay.

Range	CID positive	IFN-γ positive	CID and IFN-γ positive	CID negative	IFN-γ negative
0-10 U/L	5	2	1	46	46
10-20 U/L	12	5	2	34	44
20-30 U/L	-	-	-	2	2
30-40 U/L	1	1	1	-	-

ADA=Adenosine deaminase, bTB=Bovine tuberculosis, CID=Comparative intradermal, IFN-γ=Interferon-γ, CITT=Comparative intradermal tuberculin test

In the case of humans infected with *M. tuberculosis*, mean levels of serum ADA were also found to be significantly higher in the patients diagnosed with TB in comparison to the ones who were suffering from any other respiratory illnesses [18]. Salmanzadeh *et al*. [19] reported a higher mean serum ADA level in the human pulmonary TB group compared with patients having pneumonia, lung cancer, and control group. Mean serum ADA was 26 IU/L in PTB patients, 19.48 IU/L in patients with pneumonia, 15.8 IU/L in patients with lung cancer, and 10.7 IU/L in the control group. Afrasiabian *et al*. [20] evaluated ADA activity in 40 sputum smear-positive TB patients and 40 non-TB patients. The average (SD) of serum ADA in TB and non-TB patients was 20.88 (±5.97) and 10.69 (±2.98) U/L. The results were consistent with the findings in the present study.

Results of the present study were in contrast to the findings of Silva *et* *al*. [21] who reported that mean ADA serum in TB-positive animals was 4.45±2.33 U/L and in TB-negative animals 6.12±4.47 U/L, mean ADA values were significantly lower in serum of TB-positive animals than in the TB-negative animals.

## Conclusion

Based on the results of this study, it can be concluded that ADA seric activity can be used as a supportive surrogate marker for the diagnosis of bTB, as increase in ADA seric activity has also been reported in diseases other than bTB, in which cellular immunity is stimulated. Larger studies must be done for further validation.

## Authors’ Contributions

DC conceptualized the aim of the study, designed, planned and supervised the experiments, and corrected the manuscript. NKD performed the γ-IFN assay and Adenosine deaminase assay. NKD, GF and STS performed the CITT test. DN helped with the IFN-γ assay. MC revised the manuscript. All the authors have read and approved the final manuscript.
